# Trachoma Prevention Practice and Associated Factors in Rural Lemo District, Southern Ethiopia, 2021

**DOI:** 10.4314/ejhs.v33i1.16

**Published:** 2023-01

**Authors:** Mulunesh Girma Shobiso, Mohammed Seid Hussen, Minychil Bantihun Munaw, Mikias Mered Tilahun

**Affiliations:** 1 Wachemo University Nigest eleni Mohamed comprehensive hospital; 1 Department of Optometry, School of Medicine, University of Gondar, Comprehensive Specialized Hospital, Gondar, Ethiopia

**Keywords:** Trachoma, Prevention, Control, Epidemiology, Ethiopia

## Abstract

**Background:**

Trachoma is a leading cause of preventable blindness. It is more prevalent in areas where there is poor personal and environmental sanitation. Implementing a SAFE strategy will reduce the incidence of trachoma. The purpose of this study was to look into trachoma prevention practices and associated factors in rural Lemo, South Ethiopian communities.

**Methods:**

We conducted a community-based cross-sectional study in the rural Lemo district of south Ethiopia, covering 552 households, from July 1 - July 30, 2021. We used a multistage sampling technique. Seven Kebeles were selected using a simple random sampling method. Then, a systematic random sampling procedure with a five-interval size was applied to select the households.

Our study assessed the association between the outcome variable and explanatory variables using binary and multivariate logistic regressions. The adjusted odds ratio was calculated, and variables with a p-value below 0.05 at the 95% confidence interval (CI) were considered statistically significant.

**Results:**

The study found that 59.6% (95% CI: 55.5%–63.7%) of participants had good trachoma prevention practices. Having a favorable attitude (odds ratio [AOR]: 1.91, 95% CI: 1.26–2.89), receiving health education (AOR: 2.16, 95% CI: 1.46–3.21), and obtaining water from a public pipe (AOR: 2.48, 95% CI: 1.09–5.66) were significantly associated with good trachoma prevention practice.

**Conclusions:**

Fifty-nine percent of the participants had good prevention practices for trachoma. Health education, a favorable attitude, and a water source from public pipes were variables associated with good trachoma prevention practice. Improving water sources and disseminating health information are vital to increasing trachoma prevention practices.

## Introduction

Trachoma is one of the preventable neglected tropical diseases (NTDs) caused by *Chlamydia trachomatis* infection (1, 2). A recurring episode of infection causes abnormal scarring of the eyelid, resulting in Trachomatous trichiasis (TT), which causes permanent visual impairment and even blindness. It can be transmitted from person to person by sharing fomites, hands, flies, or bedding (3–5). The World Health Organization (WHO) reports that trachoma is a public health issue in at least forty-four countries, and nearly two million people are blind, which constitutes about 1.4% of all blindness (3, 5). Trachoma is more prevalent in Africa, followed by Asia and Central and South America (3, 6).

The Southern Nations Nationalities and Peoples' Region (SNNPR) is Ethiopia's most active trachoma region (35.8%). In SNNPR (10 and 11), the Hadiya Zone had the greatest prevalence of active trachoma (45%).

Trachoma-related blindness resulted in an estimated annual loss of eight billion United States dollars and had a high economic impact on the community (3).

People with poor personal hygiene and environmental sanitation most commonly contract trachoma. Despite Trachoma being eliminated from North America and Western Europe, it is still prevalent in Africa (1, 14, 18, 19). In addition, about half of the households in sub-Saharan Africa had access to better sanitation, and more than a quarter did not use latrines properly (13). This contributes to the high burden of this blinding condition.

Health education, attitude, and knowledge regarding trachoma prevention practices are all factors that affect trachoma prevention practices, along with socioeconomic status, the quantity of water available to the community, the time required to fetch water from its source, and other factors (1, 14–17). Even though, there is sufficient evidence on trachoma prevalence and risk factors, we know little about trachoma prevention practices in Ethiopia, especially in the study area. Therefore, understanding the current practice of trachoma prevention and exploring the factors associated with trachoma prevention play a critical role in the prevention of blindness caused by trachoma.

## Methods

**Study design and period**: A community-based cross-sectional study was conducted within the Lemo district from July 1^st^ to July 30, 2021.

**Study area**: We conducted the study in Lemo district, one of 11 districts found in the Hadiya Zone in south-central Ethiopia. The district is located 230 kilometers from Ethiopia's capital city, Addis Ababa. Based on the district administration statistics for 2020, there are 36 kebeles, 33 rural areas, and 3 urban areas. Its total projected population is 183,912, with 90,971 men and 92,941 women. One comprehensive specialized hospital offers eye care services along with seven governmental health centers, thirty-three health posts, and seventeen privately run clinics.

**Study population**: household heads with at least one child under ten who lived in Lemo district during the data collection period, Hadiya Zone, South Ethiopia.

**Exclusion criteria**: household heads were unable to respond to the questionnaire because of mental illness, other serious illnesses, and hearing problems.

**Sampling technique and procedure**: We used a multistage sampling technique during the sampling process. Based on information obtained from the district administration office, 7 kebeles (2636 households) were randomly selected out of 33 kebeles. The proportional sample size was determined for each kebele based on the number of households that were found in that kebele.

Every household within the kebele of choice was assigned a unique identification code. Finally, the households were selected using a systematic random sample procedure with a 5-interval size.

The following operational definitions are used.

**Good trachoma prevention practice**: an individual who scored eight or more on the median scale out of 13 practice measuring questions (14).

**Good Knowledge**: an individual who scored a median score of sixteen or above on twenty-five knowledge-based questions (14).

**Favorable attitude**: those who scored a median score of seven or above on nine questions measuring attitude. (14).

**Facial cleanliness**: an absence of ocular discharge, nasal discharge, and flies on the eye at the time of examination (13, 28).

**Ocular discharge**: is any discharge or sleeping sign around and/or in the eye at the time of examination (13).

**Nasal discharge**: any discharge observed in the nose at the time of examination (13).

**Fly on the eye**: at least one fly contacted the eyelid margin during eye observation (17).

**Time to a water source**: a round trip time is taken to fetch/collect water from the source (13).

**Adequacy/amount of water**: a person uses about twenty liters of water per day for domestic and personal hygiene (26).

**The cleanliness of a compound**: The absence of feces, animal dung, or/and domestic wastes in the residential compound.

**Health education**: had education on trachoma and trachoma prevention practice at least once in the previous two years.

**Use of waste disposal pit**: the presence of discarded unwanted agricultural and domestic products in the pits or ashes (the burned sign of waste).

**The cleanliness of the latrine:** absence of human excreta, and any unwanted trash on the floor of the latrine.

**Latrine use**: use of latrine for the excretion of feces and urine (29).

**Kebele**: lower-level governmental administration tier in the country.

**Data collection tool and procedure**: We adapted the questionnaires from various literature sources regarding trachoma prevention practices and associated factors. Two ophthalmic nurses and three integrated eye care workers (IECW) collected data using the Hadiyisa version, which has been translated from the English version and retranslated back into English by a language expert to maintain consistency.

It contains socio-demographic information, knowledge, attitude, and other factors related to trachoma prevention. Observations were made regarding the latrines, the cleanliness of the latrines, cleanliness of the children's faces, the availability and use of a waste disposal pit, the presence of hand washing facilities, and the cleanliness of the compound. An assessment was made of how clean the child's face was, as part of a preventive practice for adults.

**Data quality control**: A two-day training session was administered for data collectors. The data collectors were supervised by the principal investigator, and the forms were checked for completeness. A pre-test was performed on 5% of participants in one kebele with thirty households that were excluded from the study. According to the results of the pre-test, a reliability test (Cronbach's alpha) was performed for knowledge (0.93) and attitude (0.81) questions.

**Data processing and analysis**: A Kobo toolbox was used to collect the data, and the data were cleaned and export into Excel to minimize errors and incompleteness. Finally, exported to SPSS version 26 (statistical packages for social science) for analysis. Variables were presented in different tables and summaries. A binary and multivariate logistic regression were run to assess the strength of the association between each independent variable and the outcome variable. The fit of Hosmer and Lemeshow was evaluated.

There was no evidence of multi-collinearity with variance inflation factors less than one plus five for all independent variables. The crude odd ratio (COR), the adjusted odds ratio (AOR), and a 95% confidence interval (CI) of 0.05 were used to interpret the results of the final multivariable regression tables for assessing the strength of the association between the outcome and independent variables.

**Ethical considerations**: We obtained ethical clearance from the Institutional Review Board (IRB) of the University of Gondar (UoG), College of Medicine and Health Science (CMHS). Participants gave informed verbal consent after we exchanged formal correspondence with the district administration in Lemo. All responders had the option to discontinue their involvement, and we avoided personal identification.

## Results

**Socio-demographic characteristics of study participants**: The study was responded to by 552 study participants, which had a response rate of 94.4 percent. The median age was 37 years, with an inter quartile range of 14.75 years. Three hundred eight were female respondents (55.8%), and about 520 were married respondents (Table 1).

**Table 1:** Socio-demographic characteristics of study participants in rural Lemo district community, South Ethiopia, 2021 (n=552)

Characteristic	Number	Percent
Sex		
Female	308	55.8%
Male	244	44.2%
Age		
<=30	138	25.0%
31–37	147	26.6%
38–45	139	25.2%
>45	128	23.2%
Religion		
Protestant	340	61.6%
Orthodox	116	21.0%
Muslim	82	14.9%
Others	14	2.5%
Marital status		
In marriage	520	94.2%
Not in marriage	32	5.8%
Educational status		
No formal education	66	12.0%
Formal education	486	88.0%
Occupational status		
Housewife	132	23.9%
Farmer	264	47.8%
Merchant	87	15.8%
Daily labor	40	7.2%
Government employee	29	5.3%
Family size		
<=5	252	45.7%
>5	300	54.3%
Household monthly income level		
<3000 ETB	260	47.1%
>=3000 ETB	292	52.9%

Others* (only Jesus, Catholics)

**Environmental factors towards trachoma prevention practice**: Of the total study participants, 94.4% (521) got water from a public pipe; 67.3% (377) traveled more than 30 minutes to get water from its source; and 95.1% (521) consumed less water than recommended for a daily adequate supply (Table 2).

**Table 2:** Environmental Factors towards trachoma prevention practice in rural Lemo district community, Lemo district, South Ethiopia, 2021 (n=552)

Characteristic	Number	Percent
Source of water		
Public pipe water	521	94.4%
Spring water	31	5.6%
Time to fetch water from its source		
<=30 min	175	31.7%
>30 min	377	68.3%
Amount of water per person per day used		
<=20 Liter	525	95.1%
>20 Liter	27	4.9%
Sources of energy to cook food		
Electricity		
Yes	18	3.3%
No	534	96.7%
Wood		
Yes	537	97.3%
No	15	2.7%

**Trachoma prevention practice-related factors**: Health education was provided to more than half of the study participants 56.7% (313), and we observed favorable attitudes toward trachoma prevention in 52.4% (289) (Table 3).

**Table 3:** Trachoma prevention practice-related factors, rural Lemo district community, South Ethiopia, 2021 (n=552)

Characteristic	Number	Percent
Knowledge		
Good	323	58.5%
Poor	229	41.5%
Attitude		
Favorable	289	52.4%
Unfavorable	263	47.6%
Health Education		
Yes	313	56.7%
No	239	43.3%

Trachoma prevention practice: Among those respondents, 59.5% (329) had good trachoma prevention practices (95% CI: 55.5%–63.7%). The median trachoma prevention practice was 8.0 in Lemo district, South Ethiopia (n = 552). Four hundred seventy-eight (86.6%) participants were treated with antibiotics during a mass drug administration campaign, and 54.7% (302) participants used soap for face washing.

Alternatively, only sixteen respondents (2.9%) had access to hand washing facilities near the latrine. Moreover, more than 287 (52.0%) of the children did not exhibit ocular discharge, nasal discharge, or flies on the eye at the time of examination, which arose from a favorable attitude towards prevention, even if there was a limited plausible environment. Also, 37.2% (209) of the participants had a clean environment at the time of examination (Table 4).

**Table 4:** Trachoma prevention practice in rural Lemo district community, South Ethiopia 2021 (n=552)

Variables	Number	Percent
Took mass drug administration	478	86.6
Yes		
No	74	13.4
Using soap for face washing		
Yes	302	54.7
No	250	45.3
Didn't share a fomite with family	212	38.4
Yes		
No	340	61.6
Child's facial cleanliness (at		
least at one child)	287	52.0
Yes		
No	265	48.0
Flies around child's face (at		
least at one child)	211	38.2
Present		
Absent	341	61.8
Availability of waste		
disposal pits	158	28.8
Yes		
No	394	71.4
Use of waste pits		
Yes	153	27.7
No	399	72.3
Use of latrine		
Yes	527	95.5
No	25	4.5
Cleanliness of the latrine		
Yes	224	40.6
No	328	59.4
Hand washing facility near a		
latrine	16	2.9
Yes		
No	536	97.1
Hand washing practice after		
using a latrine	214	38.8
Yes		
No	338	61.2
Separated houses with		
human and animal dwelling	301	54.5
Yes		
No	251	45.5
Cleanliness of compound		
Yes	209	37.9
No	343	62.1

**Factors associated with trachoma prevention practices among study participants**: The odds of trachoma prevention practices were 2.16 times higher in communities that received health education than in those that did not receive health education. Respondents who have a favorable attitude were 1.91 times more likely to have good trachoma prevention practices than those with an unfavorable one. Respondents who received water from public pipes were 2.48 times more likely to have good trachoma prevention practices compared to their counterparts (Table 5).

**Table 5:** Logistic regression analysis of factors associated with trachoma prevention practice in rural Lemo district community, South Ethiopia, 2021 (n=552)

Characteristic	Trachoma prevention practice		COR (95% CI)	AOR (95% CI)	P-Value
				
	Good (%)	Poor (%)			
Sex					
male	146 (59.8)	98 (40.2)	1.02 (0.72–1.43)	1.44 (0.91–2.27)	0.12
female	183 (59.4)	125 (40.6)	1	1	
Age					
<=30	81 (62.3)	49 (37.7)	1	1	
31–37	92 (62.6)	55 (37.4)	0.92 (0.57–1.49)	1.05 (0.61–1.81)	0.86
38–45	90 (64.7)	49 (35.3)	1.01 (0.62–1.66)	0.84 (0.46–1.55)	0.58
>45	58 (45.3)	70 (54.7)	0.46 (0.28–0.75)	0.55 (0.27–1.13)	0.11
Marital status					
In marriage	313 (60.1)	207 (39.9)	1.51 (0.74–3.09)	1.25 (0.56–2.81)	0.59
Not in marriage	16 (50)	16 (50)	1	1	
Educational status					
No formal education	25 (37.8)	41 (62.2)	1	1	
Formal education	304 (62.5)	182 (37.5)	2.74 (1.61–4.66)	1.59 (0.83–3.05)	0.16
Occupational status					
Housewife	80 (60.6)	52 (39.4)	1	1	
Farmer	158 (59.8)	106 (40.2)	0.97 (0.63–1.49)	0.82 (0.52–1.31)	0.41
Merchant	50 (57.4)	37 (42.6)	0.88 (0.51–1.52)	0.79 (0.43–1.45)	0.45
Daily labor	24 (60)	16 (40)	0.96 (0.47–2.01)	0.79 (0.35–1.75)	0.56
Government employee	17 (58.6)	12 (41.4)	0.92 (0.41–2.09)	0.90 (0.36–2.15)	0.78
Family size					
<=5	165 (65.4)	87 (34.6)	1.57 (1.11–2.22)	1.21 (0.81–1.81)	0.36
>5	164 (54.6)	136 (45.4)	1	1	
Income level					
<3000 ETB	160 (61.5)	100 (38.5)	1	1	
>=3000 ETB	169 (57.8)	123 (42.2)	0.86 (0.61–1.21)	0.85 (0.59–1.24)	0.40
Attitude					
Favorable	204 (70.5)	85 (29.5)	2.65 (1.87–3.76)	1.91 (1.26–2.89)	0.002
Unfavorable	125 (57.8)	138 (42.2)	1	1	
Knowledge					
Good	220 (68.1)	103 (31.9)	2.35 (1.66–3.34)	1.28 (0.83–1.96)	0.25
Poor	109 (47.5)	120 (52.5)	1	1	
Source of water					
Public pipe water	318 (61)	203 (39)	2.85 (1.34 –6.07)	2.48 (1.09–5.66)	0.03
Spring water	11 (35.4)	20 (64.6)	1	1	
Time to fetch water					
<=30 min	113 (64.5)	62 (35.5)	1.36 (0.94–1.97)	1.05 (0.69–1.58)	0.83
>30 min	216 (57.3)	161 (42.7)	1	1	
Amount of water					
<=20 liters	313 (59.6)	212 (40.4)	1	1	
>20 liters	16 (59.2)	11 (40.8)	0.99 (0.45–2.17)	1.15 (0.47–2.82)	0.76
Health education					
Yes	221 (70.6)	92 (29.4)	2.91 (2.05–4.14)	2.16 (1.46–3.21)	≤0.001
No	108 (45.1)	131 (54.9)	1	1	

## Discussion

An analytical cross-sectional study was conducted to evaluate trachoma prevention practice and associated potential variables. The study results showed that 59.60% (95% CI: 55.5%–63.7) of the participants took good prevention measures against trachoma. This is marked by acceptable latrine use (95.5%), mass drug administration (86.6%), the absence of flies around children's faces (61.8%), the use of soap for face washing (54.7%), facial cleanliness (52%), and household dwelling proximity with animals (54.5%).

The trachoma prevention practice in our study was higher than those conducted in Vietnam (54.7%) (19), Oromia Region (51.5%) (15), and Tigray Region (35.6%) (14). As a result of the positive socioeconomic development of the country (30), changes in the water infrastructure, latrine use, and health care infrastructure, in comparison with previous studies conducted at an earlier time, and the smaller sample size (15), might contribute to the discrepancy.

While the cleanliness of the compound (37.1%), hand washing facility near a latrine (2.9%), latrine cleanliness (40.6%), hand washing practice after using a latrine (30.8%), and availability of a waste disposal pit (28.8%) were relatively low. To increase prevention practices for this blinding ocular condition, we should make improvements to those variables.

An important finding of this study was that a positive attitude towards trachoma prevention practices was significantly associated with a positive prevention outcome. Those who had a good attitude had a positive view of environmental sanitation and personal hygiene practices in the community, which resulted in good trachoma prevention practices as evidenced by studies (1, 14, 31, 32). Overall, communities with a good attitude had good practices for trachoma prevention. It is crucial to implement future interventions that will improve community attitudes towards prevention in order to reduce the burden of this disease.

A health education program can improve cultural perception by mitigating misunderstandings and encouraging the community to use water for personal hygiene and environmental sanitation (1, 4, 14, 33). Study subjects who had received health education had good trachoma prevention practices. Investing in health education could be an effective strategy to reduce the burden of trachoma in the community.

Trachoma prevention practice was significantly better among participants who got water from public pipes compared to participants who got water from springs. Infection contamination risks from spring water sources (13, 24, 34, 35) may cause them to be less motivated toward trachoma prevention practices. Securing a clean water supply is vital to improving trachoma prevention practices.

There was no significant association found between knowledge and trachoma prevention practice (14). Differences in study design, population, and methods have been cited as possible explanations.

It is advisable to take action to make people aware of trachoma prevention practices through health education on the importance of keeping animals and humans in a separate house, proper waste disposal, face washing, latrine use, cleanliness, and sanitary facilities near latrines.

The provision of safe water should be critical to the improvement of prevention practices. Finally, this study will help initiate successful public intervention to prevent this blinding ocular disease.

Because our study was a cross-sectional assessment of the outcome variable and could not account for time change of the potential variable, longitudinal studies in the coming years would be beneficial. Using structured quantitative questionnaires leads to not being able to glean all the information we need; it will be more comprehensive in future studies to add qualitative tools.

We adapted our questionnaires from different publications, which affected the measure of different variables, so future studies are likely to consider using standard questionnaires to enhance quality. In addition, our study was limited to one district due to resource constraints, so studies on a larger scale are anticipated in the future.
